# Frequency distribution of the hereditary Alzheimer’s disease-related genes seems to fit Poisson distribution, why?

**DOI:** 10.1038/s41421-022-00444-9

**Published:** 2022-07-28

**Authors:** Shufei Ge, Mingliang Cai, Gang Pei

**Affiliations:** 1 Institute of Mathematical Sciences, ShanghaiTech University, Shanghai, China; 2 Department of Mathematics, University of Miami, Coral Gables, FL USA; 3 Shanghai Key Laboratory of Signaling and Disease Research, School of Life Sciences and Technology, Tongji University, Shanghai, China; 4 State Key Laboratory of Cell Biology, Center for Excellence in Molecular Cell Science, Shanghai Institute of Biochemistry and Cell Biology, Chinese Academy of Sciences, Shanghai, China

**Keywords:** Bioinformatics, Molecular biology

**Fig. 1:**
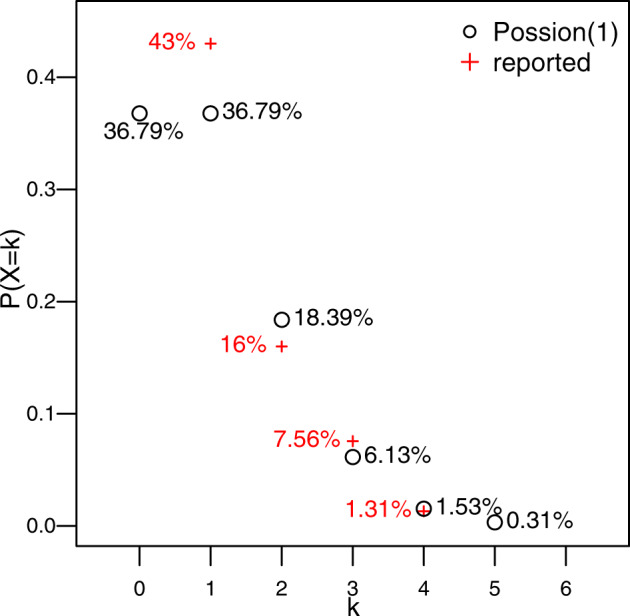
Reported percentages of level 1 (*PSNE1*, 43%), level 2 (*APP*, 16%), level 3 (*PSNE2*, *APOE4*, on average 7.56 %), and level 4 (the average of *TERM2*, *SORL1*, *ABCA7*, 1.31%) vs probabilities of Poisson distribution with the mean of 1. Here the frequency of Poisson distribution corresponds to the level of the EOAD gene mutation.

Dear Editor,

We find, much to our surprise, that the percentage of mutation or duplication related to the early-onset Alzheimer’s disease (EOAD, the well-established hereditary disease) reported in the World Alzheimer’s Disease Report 2021^1^ seems to fit a certain pattern. According to the report, *PSEN1* mutation accounts for the majority of EOAD gene mutations (43%), frequency of APP mutation represents 16%, and those of *PSEN2* and *APOE4* mutations are 6% and 9.12%, respectively, with an average of 7.56%. The next level is the rare mutations in genes *TREM2*, *SORLI*, *ABCA7* accounting for 1.17%, 1.42%, 1.33%, respectively^1–3^, with an average of 1.31%. Now we can see that the reported percentages of the hereditary EOAD gene mutations with four different levels follow a similar pattern to Poisson distribution when the mean equals 1 (Fig. 1). In addition, we performed a goodness-of-fit test for the Poisson distribution hypothesis. With the total sample size of 170^2^, the *P*-value of the test is 0.2415, which corroborates that the distribution of the reported EOAD-related mutation frequency fits a Poisson distribution with the mean being equal to 1.

An early study reports that the mutation frequencies in Big Blue mice also fit a Poisson distribution^4^. The number of emerging mutations per cell is also roughly Poisson distributed^5^. Hence, we wonder whether Poisson distribution is intrinsic to the ‘frequency’ distribution of EOAD-related mutations, or to that of any hereditary disease-related mutations. Or might it be true that Poisson distribution models frequency distribution of the gene mutations for any certain genotypes?

Interestingly, it is known that there are dozens of EOAD-related genes. We would like to hypothesize that there are some gene mutations occurring with even lower frequencies that might be close to level 5, namely ~0.31%, as predicted by Poisson distribution.

More intriguingly, the apparent fitness may provoke us to inquire into the biological meaning of the level 0. We do not know the answer at present, but we can still postulate that the level 0 could be a collection of many minor hereditary gene mutations, or the epigenetic modifications with hereditary properties, or even other hereditary risk factors unbeknownst to us yet, or the totality of the above.

We may take liberty to further speculate that for any tetraploid organism (human being is diploid), the relevant frequency would follow Poisson distribution with the mean of 2 (hexaploidy = 3, octoploid = 4, etc.).

Therefore, we would like to bring our hypothesis to community’s attentions and hopefully to find more supporting or refuting evidence.
